# Molecular Mechanisms of AKI in the Elderly: From Animal Models to Therapeutic Intervention

**DOI:** 10.3390/jcm9082574

**Published:** 2020-08-08

**Authors:** Barbara Infante, Rossana Franzin, Desirèe Madio, Martina Calvaruso, Annamaria Maiorano, Fabio Sangregorio, Giuseppe Stefano Netti, Elena Ranieri, Loreto Gesualdo, Giuseppe Castellano, Giovanni Stallone

**Affiliations:** 1 Nephrology, Dialysis and Transplantation Unit, Department of Medical and Surgical Sciences, University of Foggia, Viale Pinto Luigi 251, 71122 Foggia, Italy; barbarinf@libero.it (B.I.); madio.desy@gmail.com (D.M.); annamaria.maiorano@gmail.com (A.M.); fabiosangregorio@libero.it (F.S.); giovanni.stallone@unifg.it (G.S.); 2 Nephrology, Dialysis and Transplantation Unit, Department of Emergency and Organ Transplantation, University of Bari, 70124 Bari, Italy; rossana.franzin@uniba.it (R.F.); loreto.gesualdo@uniba.it (L.G.); 3 Nephrology, Dialysis and Transplantation Unit, Department of Biomedical Sciences, University of Foggia, 71122 Foggia, Italy; martinacalvaruso@gmail.com; 4 Clinical Pathology, Department of Surgical and Medical Sciences, University of Foggia, Viale Pinto Luigi 251, 71122 Foggia, Italy; giuseppestefano.netti@unifg.it (G.S.N.); elena.ranieri@unifg.it (E.R.)

**Keywords:** acute kidney injury, elderly, risk factors, mortality, intensive care, renal replacement therapy, kidney aging, tubular senescence

## Abstract

Acute kidney injury (AKI), a critical syndrome characterized by a sudden reduction of renal function, is a common disorder among elderly patients particularly in Intensive Care Unit (ICU). AKI is closely associated with both short- and long-term mortality and length of hospital stay and is considered a predictor of chronic kidney disease (CKD). Specific hemodynamic, metabolic, and molecular changes lead to increased susceptibility to injury in the aged kidney; therefore, certain causes of AKI such as the prerenal reduction in renal perfusion or vascular obstructive conditions are more common in the elderly; moreover, AKI is often multifactorial and iatrogenic. Older patients present several comorbidities (diabetes, hypertension, heart failure) and are exposed to multiple medical interventions such as the use of nephrotoxic contrasts media and medications, which can also trigger AKI. Considering the emerging relevance of this condition, prevention and treatment of AKI in the elderly should be crucial in the internist and emergency setting. This review article summarizes the incidence, the risk factors, the pathophysiology, the molecular mechanisms and the strategies of prevention and treatment of AKI in elderly patients.

## 1. Introduction

Acute kidney injury (AKI) is one of the most serious complications of hospitalized patients, mainly in Intensive Care Unit (ICU) [1,2,3,4], still associated to unacceptable high mortality and morbidity [5,6,7]. Patients who develop AKI during hospitalization show mortality rates of about 50% that exceed 80% if renal replacement therapy (RRT) is needed [8,9].

AKI is a clinical syndrome characterized by an abrupt decrease in kidney function that develops from few h within 7 days. The Kidney Disease: Improving Global Outcome (KDIGO) Acute Kidney Injury Work Group defined AKI as an increase in serum creatinine (sCr) by ≥0.3 mg/dL within 48 h or an increase in sCr up to 1.5 times the sCr baseline within 7 days or a urine volume <0.5 mL/kg/h for 6 h [1]. KDIGO criteria also identify AKI stages (Table 1) [1,10].

Age, sepsis, major abdominal surgery and presence of comorbidities (i.e., diabetes, hypertension, chronic kidney disease (CKD)) are conditions that can predispose to renal impairment. Among them, age seems to be one of the most relevant risk factors [4,11,12].

The world population is ageing; virtually every country in the world is experiencing growth in the number and proportion of older persons in their population with an important increasing number of patients aged 80 years or over. This group of population is projected to increase more than threefold between 2017 and 2050, rising from 137 to 425 million [13]. With advancing age, patients tend to have more comorbid chronic illnesses and disabilities which are associated with a higher hospitalization rate and also a higher incidence AKI [14]. Usually the term ‘‘elderly’’ denotes broadly older individuals, but there is not universally accepted definition or cut-off age. Indeed, many authors have proposed various ages ranging from 60 years (indicated as elderly) to >80 years (indicated as very elderly) in the attempt to define the aged status [15,16,17]. Currently, it is reasonable to call “elderly” a patient with age >65 years.

Based on these observations, several authors demonstrated that older age acts as an independent risk factor for development of AKI [11,12]. Hsu et al. showed that elderly patients are at highest risk for the most severe form of AKI, namely requiring RRT, and that the incidence of AKI in the elderly increases more rapidly than in younger patients [11]. The BEST Kidney study demonstrated that older age was independently associated with a higher mortality in a large critically ill patients’ cohort with AKI [18]. Furthermore, the older patients present several comorbidities that are themselves additional risk factors for AKI development [4,19,20,21]. First, a pre-existing condition of CKD may negatively affect the risk of AKI development. A reduction of glomerular filtration rate (GFR) with alteration of hemodynamic and/or reduced renal function reserve increases the susceptibility to injury. Other comorbidities such as diabetes mellitus, hypertension and hearth failure (HF), frequently affecting elderly patients, could act as additional risk factors for AKI establishment. Moreover, older patients are exposed to multiple potentially nephrotoxic medications and invasive procedures [22,23]. The elderly exhibit, in absence of other specific comorbidities, a process of physiological, structural and functional involvement, named “kidney aging”, which represents a further risk factor for AKI [24].

Recently, several reports show that in recent years, the mean age of patients admitted to ICU has considerably increased. In western countries, elderly people, while representing the 18% of the general population, account for 45.5% of the hospital admissions and rise to 60% of the patients admitted to ICU [20]. It is estimated that almost 50% of ICU beds in the United States are occupied by patients aged ≥65 years [25].

In this review article, we will highlight the epidemiologic studies, the risk factors, the pathophysiology, the molecular mechanisms and the strategy for prevention and treatment of elderly patients affected by AKI.

## 2. Epidemiology

**Figure 1:**
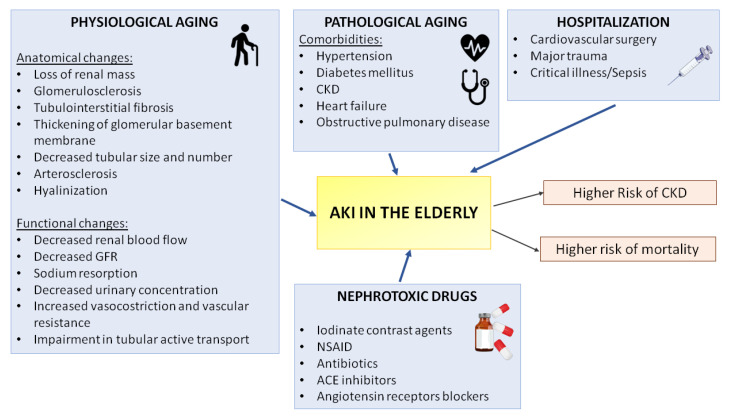
Principal risk factors of AKI in the elderly. A large collection of factors can predispose elderly to AKI. Between them, the most important are the physiological aging of the kidney that is associated to common anatomical and functional changes and the pathological aging correlated to a wide spectrum of comorbidities (such as such as hypertension, diabetes mellitus, heart disease, CKD). Other exogeneous factors include the poor/good outcome of medical interventions and the consequent prolonged hospitalization. Furthermore, nephrotoxic drugs therapies as the contrast media for radio analysis can increase susceptibility to AKI in older patients. The failure to compensate an episode of AKI can lead to higher risk to progression to CKD and depending from severity also of mortality. NSAID, Nonsteroidal anti-inflammatory drug, ACE Angiotensin-converting enzyme.

The real entity of AKI phenomenon is hard to assess due to the lack of homogeneity in definitions and diagnostic criteria. Furthermore, in the elderly people the estimate of AKI epidemiology is more difficult because of the various clinical settings and, mostly, the several indications of “elderly” population present in literature. Nash et al. [2] reported that incidence among hospitalized patients ranged between 5% and 7%, while the incidence of AKI in the general ICU population is higher, ranging between 20% and 40%. Santos and Matos [26] found that among ICU patients, those with AKI were older as compared with non-AKI patients (56.4 ± 18.8 vs. 46.8 ± 16.5 years, *p* = 0.0028). Joannidis et al. [27], comparing the RIFLE and AKIN classification criteria in a cohort of 16.784 ICU patients, demonstrated that within 48 h after admission, AKI had an incidence ranging between 28.5% and 35.5%, and the mean patient age was 63 years; moreover 25% of patients who developed AKI were 75 years old or older. Already more than 30 years ago, Groeneveld et al. [28] demonstrated that the age-related yearly incidence of AKI rose from 17 per million in adults younger than 50 years to 949 per million in the 80–89-year-old age group. More recently, Ali et al. [29] showed that the mean age of patients with AKI in a large European cohort was 76 years. Particularly, the group of older patients (more than 80.5 years) had a higher risk for adverse outcomes. More recently, Hsu et al. [11] analyzed the incidence rates of dialysis-requiring AKI in the US Nationwide Inpatient Sample, a representative dataset from 2000 to 2009. In this decade, the incidence of dialysis-requiring AKI globally increased in each stratum of age, but the absolute incidence rates of AKI were highest in elderly individuals (Figure 1).

In conclusion, even if the real nature of the AKI phenomenon in the elderly is hard to define, it is well known that AKI incidence in older patients grows yearly and that the age itself represents an independent risk factor for AKI onset.

## 3. Pathophysiology of AKI in the Elderly

**Figure 2:**
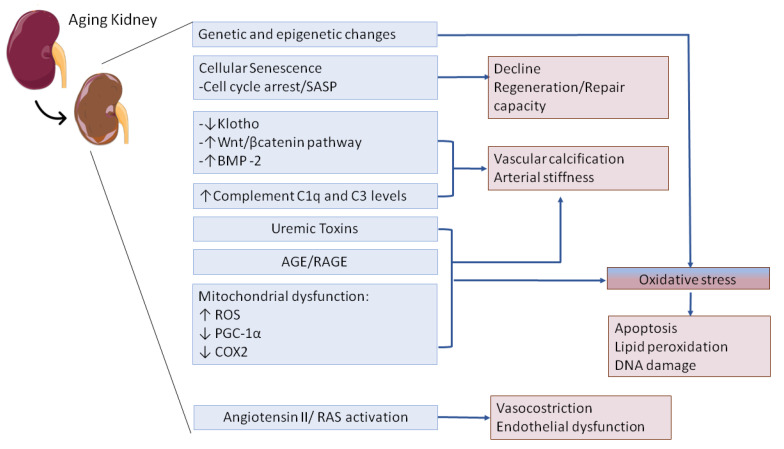
Central mechanisms underlying the aging kidney and the increased susceptibility to AKI. Several molecular mechanisms are involved in the aging kidneys from intrinsic genetic and epigenetic changes, to cellular senescence, systemic complement activation, the release of uremic toxins and AGE, the impairment of Angiotensin II/ RAS axis (indicated in blue).These events affected the renal homeostasis and the ability to response to AKI injury leading to common kidney alterations at tubular level (as the decline regeneration) and endothelial level (with the microvascular dysfunction, the arterial stiffness and the vascular calcification) (indicated in pink). Oxidative stress is a central mediator in renal aging and should be encountered as initial cause and chronic inducer of AKI in elderly by the time. Abbreviations: SASP Senescence Associated Secretory Phenotype, BMP-2 Bone morphogenetic protein 2, AGE Advanced Glycation End Products, RAGE Receptor for Advanced Glycation End Products, ROS reactive oxygen species, PGC-1α Peroxisome proliferator-activated receptor gamma coactivator 1-alpha, COX2 cytochrome c oxidase subunit 2.

Usually, AKI may be classified as pre-renal, renal or post-renal depending on the mechanism causing the injury: reduction of the renal flow (perfusion), direct renal tissue injury or urinary tract obstruction, respectively (Figure 2). The most common type of AKI, accounting for 40–60% of all causes [20,30], is the pre-renal form, also known as functional AKI, which is due to significantly lowering of renal plasma flow (RPF) and consequent reduction of the GFR, which leads to accumulation of nitrogenous products. The aging kidney is prone to pre-renal AKI, especially because of alterations in blood vessels: as kidneys get older, the vascular tone of blood vessels becomes altered, tending to favor vasoconstriction; initially, different vasodilators responses (Nitric Oxide, Angiotensin (II)) compensate for these changes, but when the kidneys are subject to an additional acute insult, these compensatory mechanism may fail [31].

The RPF reduction may be due to several causes that imply renal ischemia: loss of fluids (vomiting, diarrhea and diuretics), lower cardiac output (such as HF), drugs inducing impairment of renal autoregulation (Nonsteroidal anti-inflammatory drugs, NSAID, angiotensin-converting enzyme inhibitors ACEi, angiotensin receptor blockers). Generally, this condition is reversible if the triggers are removed. A dysfunction of the renal parenchyma causes renal AKI, defined as structural AKI. Commonly, the first cause of renal AKI is the acute tubular necrosis (ATN), with an incidence >70% [32,33].

It might have ischemic or toxic etiology and could follow a persistent pre-renal form. Other disorders leading to AKI include vascular pathologies (i.e., venous or arteriosus thrombosis, cholesterol embolization), tubulointerstitial nephritis (10–20%), infections or medications, glomerulonephritis and cortical necrosis (1–10%) [20]. The post-renal or obstructive AKI is the less frequent form of AKI (2–4%) in the general population, but it is more common in the elderly (10%). Usually, it is due to obstruction of the urinary tract caused by kidney stones, tumors, prostatic hypertrophy or retroperitoneal fibrosis [20,34].

## 4. Risk Factors for AKI Development in the Elderly

### 4.1. Kidney Aging

Despite the absence of other specific nephropathies, the renal tissue in elderly patients physiologically meets a “kidney senility” or “kidney aging”, characterized by structural and functional alterations [24,35,36]: decreased renal mass, reduced number of functional glomeruli (glomerulosclerosis) associated with a compensatory glomeruli hypertrophy, atherosclerosis, interstitial fibrosis, fibro-intimal hyperplasia (due to thickening of internal elastic lamina) and hyalinization. These senescence-related phenomena significantly worsen renal mass and renal blood flow (RBF), thus reducing kidney self-renewal capacity and facilitating the injury onset. It is estimated that RBF decreases >10% every decade of life. Consequently, in the attempt to maintain normal GFR, a compensatory glomerular hypertrophy is established, followed by increased filtration fraction and renal vasoconstriction [21,37,38,39]. Kidney aging induces a reduction in the mitochondrial energy production, with impairment in tubular active transport. This tubular injury might be detected by impairment in reabsorption of glucose and increase of urinary proteins [24,37]. Furthermore, aging implies an enhancing of the intra-renal cellular apoptosis rate, leading to a lowering of functional nephrons. Moreover, the elderly seem to show a lower cell proliferation rate, which in turn reduces the regeneration response after kidney damage [21,24,37,38]. In addition, the reduced nephron number also induces alterations in sodium homeostasis [21], thus impairing the urine concentration process. Taken together, these senescence-related modifications lead to larger volume depletion and urine dilution, which can ease dehydration status.

In the elderly population, dehydration may be due to several pathological conditions: Diarrhea, vomiting and abundant sweating are the most frequent. In particular, aged patients with dementia can have feeding and swallowing difficulties (dysphagia) with serious concerns for intake of fluids and malnutrition [40,41]. If these noxae overlap an older patient with senescence-related conditions (decreased capacity of urinary concentration, impaired hypothalamic regulation of the thirst sense, hypoproteinemia and the altered distribution of body fluids), the risk for dehydration development is much higher [19] (Table 2).

### 4.2. Comorbidities

The older patients present several comorbidities increasing the susceptibility to kidney injury. Among them, hypertension, diabetes mellitus, heart disease and pre-existing chronic kidney disease (CKD) are the most frequent. Hypertension affects more than 50% of the general elderly population. Continued high blood pressure produces increased vascular hydrostatic pressure with consequent thickening of arterial wall, due to increased smooth muscle and hyaline content of the media, and stenosis of the lumen, which in turn induces reduction of the RBF. These alterations make the kidney more prone to develop pre-renal AKI in case of hypo-perfusion [21,37,42,43]. Moreover, hypertensive patients develop atheromatous plaques, thus reducing the vascular lumen and the parenchymal perfusion and altering the Renine-Angiotensine-Aldosterone System (RAAS). As a result, increased arterial stiffness and renal vascular resistance (RVR) are observed and, if hypoperfusion occurs, the risk of AKI onset is much higher. Chronopoulos et al. showed that the RAAS of the elderly population functionality decreased by 30–50%, if compared with younger control [37].

Diabetes mellitus is present in almost 20–30% of the elderly population in Western countries [44]; the high-glucose concentration damages blood vessels (microvascular damage) also in the glomerular structure, leading to several toxic effects and consequent micro-infarcts with reduced renal functional reserve. The persistence of hyperglycemia induces the formation of advanced glycation end-products (AGE), which lead to increased production of extracellular matrix, occlusion of the glomerular capillaries and cellular damage [23,42].

Current data suggest that a higher AKI risk, AKI-morbidity and mortality incidence is observed in the presence of type 2 diabetes mellitus or in other diabetic disease.

Older individuals with diabetes are a group at particular risk of AKI due to multiple risk factors such as presence of other co-morbidities including CKD and predisposition to serious infections [45]. In a prospective case-control study of older adults, Mittalhenkle A et al. evaluated the AKI incidence in a cohort of 5731 older individuals and found a significant association of diabetes mellitus, hypertension, and current smoking with incident acute renal failure (AKI) [46]. The relevant correlation between AKI epidemiology and outcomes in diabetes mellitus, together with the recommendations on blood glucose control have been reviewed elsewhere [33,47].

Elderly patients frequently develop cardiopathies with heart failure (HF), which accounts almost 20% of hospital admissions [48]. An impaired cardiac function induces reduction of cardiac output and hypoperfusion of the peripheral tissues, due to increased pulmonary and venous systemic pressures despite a normal venous return. If the cardiac function abruptly worsens (i.e., acute myocardial infarction), renal perfusion further decreases, causing AKI [48,49].

The rapid worsening of cardiac function leading to AKI is commonly defined as cardiorenal syndrome (CRS) and causes high mortality rates. Elderly patients may be more susceptible to CRS not only for comorbidities such as hypertension or HF, associated with poor prognosis but also for the drugs for HF that can significantly impact the clinical outcomes.

In this retrospective study, Hu W et al. analyzed the incidence, risk factors and prognosis of cardiorenal syndrome type 1 (CRS1) in elderly patients [50] and found that incidence of cardiorenal syndrome type 1 was higher compared to younger cohort of patients (52.56% vs. 25.9–38.9%) [51]. Interestingly, also the use of diuretics together with the reduced eGFR were associated with the higher risk factors of CRS1 in patients, while the use of diuretics, beta-blockers and dialysis during hospitalization were predictors of in-hospital mortality.

Although several studies highlighted the higher prevalence of CRS in geriatric patients, the complex area exploring tailored treatments for elderly and based on eGFR, nutrition station or the use of diuretics deserves more investigation.

Shirakabe et al. recently demonstrated that serum heart type fatty acid binding protein (s-HFABP—A cardiac biomarkers of acute HF) levels were significantly higher in patients with worsening renal failure [52]. Usually, renal impairment has been attributed to hypoperfusion of kidney, due to progressive impairment of cardiac output or intravascular volume depletion; nevertheless, attention has shifted from cardiac output to venous congestion as the most important hemodynamic determinant: Increased renal interstitial pressure is an important mechanism of renal failure in patients with acute HF [52].

A large collection of factors can predispose the elderly to AKI. Between them, the most important are the physiological aging of the kidney that is associated to common anatomical and functional changes and the pathological aging correlated to a wide spectrum of comorbidities (such as hypertension, diabetes mellitus, heart disease and CKD). Other exogeneous factors include the poor/good outcome of medical interventions and the consequent prolonged hospitalization. Furthermore, nephrotoxic drug therapies such as the contrast media for radio analysis can increase susceptibility to AKI in older patients. The failure to compensate an episode of AKI can lead to higher risk to progression to CKD and depending on severity also of mortality. NSAID, Nonsteroidal anti-inflammatory drug, ACE Angiotensin-converting enzyme.

### 4.3. Polypharmacy

Commonly, the coexistence of several comorbidities in elderly patients needs multiple drugs (polypharmacy), which may be nephrotoxic alone or in combination [53]. The drugs may induce renal damage with several mechanisms, often combined, such as acute hypersensitivity, chronic accumulation and intoxication given the reduced excretion of elderly kidney.

Interestingly, there is an increased incidence of interstitial nephritis in the elderly because of polypharmacy. In particular, hypersensitivity has been observed after antibiotics treatments (i.e., penicillins, cephalosporins and sulfonamides). Recently, in a comprehensive study of a national pharmacovigilance database, gentamicin emerged as the main drug class that frequently induced AKI [54]. Aminoglycosides and amphotericin B are well recognized nephrotoxic agents in the elderly, and the estimation of appropriate dosing by careful evaluation of creatinine and body mass is a crucial issue for clinicians [23].

As reviewed elsewhere, drug-related renal damage may be both dose-dependent and time-dependent. Normally, for the antibiotics, the onset of AKI is observed within 3 weeks of drugs initiation. Regarding the NSAID-induced AKI, it can occur after few months with symptoms that differ from the hypersensitivity reactions, are related to minimal change disease or membranous nephropathy and are characterized by proteinuria. As already discussed, renal function in the elderly is significantly affected by the loss of urinary concentrating ability, the reduced capacity to retain salt and water leading to volume depletion and dehydration and the decreased prostaglandin production. NSAIDs, commonly used by approximately 10–25% of the elderly, contribute to further inhibiting production of vasodilatory prostaglandins. Therefore, the aging-induced hemodynamic changes leading to reduction of renal plasma flow and glomerular filtrate rate [23,53,55,56], together with the NSAID increased renal vasoconstrictive response, worsen the renal exposition to nephrotoxic medications, leading to a chronic accumulation.

Furthermore, other class of drugs as ACE-inhibitors or Angiotensin Receptor Blockers are associated to intoxication due to volume depletion or underlying CKD (Figure 1).

Finally, another class of drugs that should be considered in the pain management of geriatric population is the chronic use of opioids. Clinically, in the elderly, opioids can result in AKI because of significant changes in GFR, dehydration, rhabdomyolysis and urinary retention. In patients with reduced GFR, accumulation of active metabolites due to lower renal clearance has been observed especially for morphine, pethidine and codeine leading to more side effects at CNS and respiratory depression. Therefore, the use of opiates with minimal renal excretion (e.g., fentanyl, oxycodone, hydromorphone and tramadolIn) is preferred [56,57,58].

### 4.4. Other Causes

The large use of iodinated contrast agents for diagnostic imaging [59] is responsible for the onset of contrast induced AKI (CI-AKI). This condition is one of the most frequent causes of AKI in the elderly and reaches a prevalence of the 11% in hospitalized population with an incidence of 6-14% in people aged >65 years old [60]. CI-AKI is due to intra-venous and intra-arterial infusion of contrast agents during diagnostic or invasive procedures. The iodinated contrast medium, in addition to direct toxic tubular damage, may alter the renal perfusion and the intra-renal hemodynamics by inducing hypoxic damage in the renal medullary (S3 segment of the proximal renal tubule) [59,60,61].

In particular, the incidence of CI-AKI increases to 20% to 40% in high-risk patients (diabetes, congestive heart failure, CKD with eGFR <30 mL/min and older age); moreover, intra-arterial contrast exposure has been shown to be more nephrotoxic than intravenous use, probably due to higher concentrations of radiocontrast [62].

In elderly patients, obstructive forms of AKI are very frequent. The main causes of obstruction are benign prostatic hypertrophy and prostate cancer (in males) and pelvic or retroperitoneal neoplasms. Urological cancers may worsen renal function due to both obstructive action and cancer chemotherapies, which can potentially exert nephrotoxic effects. Moreover, the increased production of pro-inflammatory cytokines [63] can also predispose to AKI development. Moreover, the urine obstruction increases the risk for urinary tract infections (UTI), which are more frequent in the elderly patient. UTI in the elderly in turn often evolve into urinary sepsis with establishment of septic shock and septic AKI, which significantly worsen hospitalization length and patient survival [64].

Sepsis is a serious medical condition characterized by a maladaptive host response to infection, leading to organ dysfunction and shock [65,66,67]. Multi-organ failure (MOF) can frequently occur in sepsis with development of AKI such as in COVID-19 associated AKI [68]; interestingly, 35–65% of ICU patients meet the diagnostic criteria of AKI, and in 40–70% of cases, AKI is attributable to sepsis. These patients in ICU with sepsis have a further increase in mortality rate [65].

Nowadays sepsis is a major cause of AKI in critically ill patients, but in older patients its impact is significantly greater [69].

## 5. Molecular Mechanisms of AKI in the Elderly: Lessons from Animal Models and Clinical Trial

The mechanisms underlying the increased susceptibility of aged kidney to AKI involve a complex interplay of processes as genetic and epigenetic changes [70], cellular senescence, oxidative stress [24] and systemic and local microenvironmental changes as complement activation or the persistent release of pro-inflammatory cytokines [71]. Many of these events result in well-known structural and functional changes in the elderly (i.e., glomerulosclerosis, interstitial fibrosis or the decline in GFR) thus leading to AKI or to further progression to CKD. Recent evidence from clinical and experimental models of biological and accelerated aging demonstrated that healthy kidney aging shows several biological pathways with CKD or I/R, sepsis, contrast agent-induced AKI. As with humans, physiologically aged mice displayed an increased AKI incidence and a decreased regeneration potential of epithelial cells after different nephrotoxic injuries as sepsis [72] or I/R [73,74]. Therefore, understanding this signaling is essential to investigating possible common therapeutic intervention strategies to delay AKI in the elderly.

Several molecular mechanisms are involved in the aging kidneys from intrinsic genetic and epigenetic changes, to cellular senescence, systemic complement activation, the release of uremic toxins and AGE and the impairment of Angiotensin II/ RAS axis (indicated in blue). These events affected the renal homeostasis and the ability to respond to AKI injury leading to common kidney alterations at tubular level (as the decline regeneration) and endothelial level (with the microvascular dysfunction, the arterial stiffness and the vascular calcification) (indicated in pink). Oxidative stress is a central mediator in renal aging and should be encountered as initial cause and chronic inducer of AKI in the elderly in time. Abbreviations: SASP, Senescence Associated Secretory Phenotype; BMP-2, Bone morphogenetic protein 2; AGE, Advanced Glycation End Products; RAGE, Receptor for Advanced Glycation End Products; ROS, reactive oxygen species; PGC-1α, Peroxisome proliferator-activated receptor gamma coactivator 1-alpha; COX2, cytochrome c oxidase subunit 2.

### 5.1. Klotho Downregulation in the Elderly

*α*-Klotho, referred to as Klotho, is the most important renal anti-aging protein expressed predominately in the tubular epithelium [75]. Klotho is encoded as a single-pass transmembrane protein representing the co-receptor for fibroblast growth factor-23 (FGF-23), a bone-derived hormone that plays a critical role in phosphate homeostasis. Klotho-deficient mice exhibit reduced lifespan, skin and muscle atrophy, osteoporosis and ectopic calcification whereas Klotho overexpression increased lifespan [76]. Therefore, Klotho is involved in vascular calcification but also cellular regeneration and senescence. Next to full-length transmembrane form, the proteolytic shedding of the extracellular domain produces the truncated, secreted form of Klotho (sKlotho) [77]. Of particular interest, secreted Klotho can exert its biologic actions in several tissues, acting as an endocrine hormone [78]. A wide spectrum of evidence describes the reduced Klotho expression in the kidney, blood and urine after I/R in mouse [79], rat [80] and swine [81] models.

In humans, both transmembrane and secreted forms of Klotho significantly decrease with age and are down-regulated in several conditions of AKI and CKD [82,83,84,85]. Strinkigly resembling a pro-aging phenotype, the Klotho loss is associated with vascular calcification and impairment [86].

In the last decades, several studies have been reported to elucidate the exact correlations between sKlotho and the renal function impairment in CKD patients. In a meta-analysis including 1457 CKD patients, Qinglian Wang et al. identified a significant positive correlation between sKlotho and eGFR. [87]. Starting from 611 studies, the authors included 9 publications of retrospective and cross-sectional analysis with mean age of CKD patients ranging from 45.75 to 68 years [88,89,90]. The meta-analysis also confirmed previous results of large cohort study that did not found significant differences of circulating Klotho between CKD stage 2, 3a, 3b, and 4 patients [91]. However, as well investigated by Scholze A. et al., CKD patients with s-klotho below 204 pg/mL had higher age, lower phosphate clearance, and lower bone-specific alkaline phosphatase, indicating that older age more than CKD stage can affect the s-Klotho concentration and function (reference Klotho level in normal adults from 239 to 1266 pg/mL) [92]. However, more data from larger prospective longitudinal studies are required to validate this hypothesis. Recently, therapies able to restore klotho levels through re-activation of endogenous Klotho or administration of exogenous Klotho have been considered as novel treatment strategy for AKI and CKD. Several approaches have been evaluated from the epigenetic approaches (such as the inhibition of Klotho gene promoter hypermethylation and histone acetylation), to Klotho cDNA delivery via viral carrier, to the administration of soluble Klotho protein that is currently under clinical trial (NCT03532568) [93,94,95].

### 5.2. Wnt/ β-Catenin Pathway Is Activated in the Elderly during AKI

Wnt/β-catenin signaling is a conserved pathway that controls early nephrogenesis and normally is kept silent in adult kidneys [96]. The signaling is re-activated during AKI and in the progression from AKI-to-CKD models, predominantly in damaged tubular epithelial cells. As Klotho levels fall during the injury, the Wnt signaling becomes activated inducing renal senescence, vascular calcification [97] and tubulointerstitial fibrosis [98]. From the other side, blockade of Wnt/β-catenin prevents AKI to CKD progression [99].

In clinical settings, in a cohort of 91 CKD patients, the Wnt/β-catenin pathway has been assessed as the major mechanism regulating the bone morphogenetic protein 2 (BMP-2) level, a central mediator that regulated the calcium deposition in arterial stiffening and vascular lesions in the elderly [97]. Specifically, BMP-2 significantly higher in CKD patient’s serum, upregulated the expression of β-catenin whereas a BMP-2 neutralization antibody reversed these effects. The link between BMP-2 and Wnt signaling is bidirectional since the knockdown of β-catenin abolished the effect of high phosphate and BMP-2 on vascular calcification [97,99].

Recently, the activation of intrarenal Wnt/*β*-catenin was assessed in a prospective, multicenter cohort study involving 721 patients with severe AKI in patients after cardiac surgery. Interestingly, the induction of Wnt/*β*-catenin evaluated by urinary matrix metalloproteinase-7 (uMMP-7) levels was a predictor for severe AKI and poor-outcomes [100].

Lately, the contribution of Wnt/*β*-catenin together with the renin-angiotensin system (RAS) to renal aging process has been highlighted [101]. RAS has been shown to promote the pathophysiological processes of various aging-related disorders, including cardiovascular diseases, CKD, dementia, osteoporosis and cancer [102]. Recent studies have demonstrated that, similarly to Klotho findings, inhibition of RAS promotes longevity in rodents and prevented the aging-related functional decline in skeletal muscle, suggesting the involvement of RAS in the aging process by down-regulation of Wnt/β-catenin signaling [103]. In accordance, in an established aging mouse model, Jinhua Miao et al. showed that the inhibition of Wnt/β-catenin signaling and the RAS could slow the onset of renal fibrosis and of age-related mitochondrial dysfunction [104]. These results pave the way for the use of angiotensin-converting enzyme (ACE) inhibitors, used to delay renal aging occurring after AKI in the elderly. However, the risk/benefit ratio of RAS blockers must be evaluated in elderly CKD patients [105]. In the last years, several Wnt antagonists, such as small molecule inhibitor ICG-001 [106] and soluble Klotho (NCT03532568) have been evaluated in various clinical trials. Targeting this signaling may hold promise for future treatment of AKI in the elderly and to slow the progression into CKD.

### 5.3. Complement System in the Elderly

Complement system, an essential player of innate immune system is recognized as pivotal in several kidney diseases from glomerulonephritis (Lupus Nephritis, C3 glomerulopathy, IgAN) to AKI and further progression to CKD [107,108].

Complement system can be activated by three pathways: the classical, the lectin and the alternative. All the three signaling led to the cleavage of C3 central component and to formation of the terminal lytic/pro-inflammatory membrane attack complex (MAC) on infected or damaged target cells [109]. In the elderly, besides renal disease, aberrant complement activation is involved in the physiopathology of several conditions such as Alzheimer’s disease [110], Amyotrophic lateral sclerosis, multiple sclerosis or age-related macular degeneration [111]. Interestingly, the classical pathway initiator C1q level increases with aging and can activate Wnt/β-catenin signaling that is involved in the skeletal muscle aging. To note, C1q has been also identified to play a role in the development of arteriosclerosis and arterial stiffening that occurs in advancing aging [112]. Originally, initial studies indicating the correlation between complement system and healthy aging were performed by Yonemasu K et al. more than 50 years ago [113]. The latter demonstrated that in a cohort of healthy volunteers (from birth up to 75 years) C1q and C3 levels changed with age. C1q increased gradually from birth to 60 years; C3 instead reached a higher level at 1 year, decreased until puberty and increased after this age. Next to the classical pathway, also the alternative pathway is upregulated in the elderly [114]. More recently, in a cohort of 120 healthy volunteers, M. Gaya da Costa et al. investigated the inter-individual variation in complement activity and the influences of age and sex. Interestingly, the authors found that classical and alternative pathways were significantly higher in the elderly, in contrast to lectin pathway activation [115]. Besides physiological and chronological aging, premature aging induced by AKI episodes could be modulated by complementary inhibition [108]. In a pig model of I/R we demonstrated that C1-INHIBITOR (C1-INH), an endogenous regulator of classical and lectin pathway, was able to protect from tubular senescence preserving the level of anti-aging protein Klotho and reducing cell cycle arrest proteins levels [70,81]. In clinical settings, several complement-blocking agents have been used to prevent complication associated to kidney transplantation, thus arresting the progression of fibrosis and the accelerated renal aging. There are ongoing clinical trials evaluating the efficacy of C1-INH [116,117,118] or Eculizumab [119] in preventing the development of I/R injury, DGF and in the prevention and treatment of antibody mediated rejection (ABMR) [120].

### 5.4. Oxidative Stress

Several experimental and clinical data support the central role of oxidative stress in the injury phase of AKI and in the acquirement of an aging phenotype. Under physiological circumstances, the metabolic homeostasis is retained by the balance between reactive oxygen species (ROS) generation and antioxidant production [121]. Oxidative stress occurs when metabolic disorders can alter this balance; thus, increased production of oxidants can lead to cellular apoptosis, lipid peroxidation, dysfunction of proteins and damage of DNA. The principal mechanism of ROS generation is the mitochondrial oxidative phosphorylation by cytochrome oxidase; other processes include the xanthine oxidase and NADPH oxidase complex, whereas the endothelial isoform of nitric oxide synthase (eNOS), and the AKI induced inducible nitric oxide synthase (iNOS) are the main source of NO production [122,123,124,125].

The kidney receives about 25% of total blood supply; for that reason, it has the second highest mitochondrial content and oxygen consumption after the heart. In particular, proximal tubules require more active transport mechanisms than other renal cell types because they reabsorb 80% of the filtrate that passes through the glomerulus, including glucose, ions and nutrients [126]. Thus, they contain more mitochondria than any other structure in the kidney and are the cells mainly affected by acute damage. Mitochondrial dysfunction has been identified as the earliest initiator of AKI, as observed in the context of sepsis and I/R [125,127] preceding also renal manifestations as the increase of serum creatinine levels. In addition, defects in the electron transport chain promote oxidative stress by electron leakage to form superoxide radicals, which play a key role in triggering cellular senescence and accelerating aging [128]. In an established mouse model of renal aging, Jinhua Miao et al. [104] demonstrated that master regulators of mitochondrial biogenesis as PGC-1α (mitochondrial biogenesis-related transcription factors peroxisome proliferator-activated receptor-C coactivator-1α), cytochrome *c* oxidase subunit 2 (COX2), complex IV subunits cytochrome *c* oxidase 1 (COX1) and the mitochondrial mass were significantly decreased in the elderly animals, especially at 24 months of age [129]. Furthermore, authors also found that inhibition of pro-aging and pro-fibrotic Wnt/β-catenin activity reduced tubular senescence and restored renal mitochondrial functions by preserving mass and diminishing the production of ROS, therefore delaying the occurrence of kidney aging. Previous studies evaluated the correlation between oxidative stress and accelerated renal aging after AKI. Martin R et al. analyzed the mRNA level of antioxidative enzymes (i.e., Cu/Zn-and Mn-SOD, catalase, GSH reductase and GSH peroxidase) in liver and kidneys of young (6 months) and old (22–25 months) rats [130]. Interestingly, authors showed that gene expression of antioxidative enzymes is affected by age; in particular, in old kidneys, the expression of enzymes progressively declined in the hypoxic and reoxygenation groups indicating relevant insights in the pathophysiology of I/R in elderly.

From the other side, cellular senescence in renal aging and oxidative stress induced by mitochondrial dysfunction may be mutually connected. In human renal tubular epithelial cells (HK2), Small DM et al. confirmed that that oxidative stress induced by moderate hydrogen peroxide (H_2_O_2_) treatment increased cell senescence as a natural mechanism to delay metabolic processes and enter in cell cycle arrest in order to avoid further oxidative damage [123]. Strikingly, they found that also cell cycle arrest induced oxidative stress at levels similar to H_2_O_2_ treatment alone. Taken together, these data indicated that oxidative stress and renal senescence are central players into the pathogenesis of CKD and should be considered as faces of the same coin.

In clinical settings, augmented level of oxidative has been detected in the elderly, in patients with progressive CKD or ESRD and in particular in diabetic nephropathy patients due by the accumulation of a type of uremic toxin formed by glycation named advanced glycation ends products (AGEs) [131,132,133]. It is well known that AGEs induce oxidative stress, accumulated with age, and are primarily excreted by a functioning kidney. A link between inflammation, oxidative stress, AGEs and CKD was shown in studies of mice demonstrating that reduction of AGE levels by drugs or decreased intake of AGEs reduces CKD and cardiovascular disease in the aging population [132,134,135]. These data support the hypothesis that AGEs in the diet are very important contributors to renal and cardiovascular lesions. AGEs signal is exerted by two receptors, one of which is anti-inflammatory (AGER1) and the other proinflammatory (RAGE) [133,136]. Unfortunately, although oxidative stress can be modulated in elderly subjects and patients with CKD stages 3–4 by a simple dietary modification that results in reduced AGEs serum and tissue level, diabetic patients have a decreased ability to metabolize and excrete oxidants prior to detect relevant changes in serum creatinine [122,134]. Therefore, in these patients, new, early, therapeutic approaches to counteract oxidative stress are required. Beside nutrition, promising therapeutic targets include uremic toxin absorbents and inhibitors of AGEs or the receptor RAGE. Probiotics and prebiotics maintain gut flora balance and also prevent CKD progression by enhancing gut barriers and reducing uremic toxin formation [133]. Interestingly, the Nuclear factor erythroid 2-related factor 2 (Nrf2) signaling has been showed to ameliorate oxidative stress but also to reduce elevated AGE levels. Nrf2 is a central transcription factor for the antioxidative stress response able to induce the expression of cytoprotective genes related to redox and detoxification. Nrf2 activity is regulated by the oxidative-stress sensor molecule Kelch-like ECH-associated protein 1 (KEAP1) [137]. Given the ability to rapidly respond to oxidative stress, the KEAP1-Nrf2 system has been proposed as a promising therapeutic target for renal damage. Recently, bardoxolone methyl, an Nrf2 activator and NF-κB suppressor, has been tested as a therapeutic agent in patients with CKD and type 2 diabetes [137,138,139].

## 6. The Diagnosis of AKI in Older Population

Serum creatinine is widely used in the assessment of renal function to detect impaired renal function. Serum creatinine is a convenient test, but it has several limitations to interpret renal function, mainly in elderly people. Glomerular filtration rate (GFR) estimation uses serum creatinine levels along with other variables, but still, it can be erroneous due to many confounding variables. First, serum creatinine levels rise when kidney damage has already occurred. Furthermore, serum creatinine levels depend on the creatinine generation rate, the volume of distribution and the removal rate [140].

Decrease in muscle mass, protein metabolism and level of hydration, often found in elderly, could affect serum creatinine levels and delay AKI recognition.

In addition, scoring systems have been developed to predict CI-AKI in patients after coronary angiography or percutaneous coronary intervention based on preprocedural characteristics for early prediction of (PCI) [141,142]. Novel markers of AKI are emerging such as neutrophil gelatin-associated lipocalin (NGAL), cystatin C, interleukin 18 (IL-18), kidney injury molecule 1 (KIM-1), L-type fatty acid binding protein (L-FABP), netrin 1, N-acetyl-beta-D-glucosaminidase (NAG), alfa1-macroglobulin. The use of these novel biomarkers looks forward to an earlier detection of AKI and a prompt institution of preemptive measures. At present, only cystatin C has been validated as a reliable biomarker of AKI; moreover, a combination of both serum creatinine and serum cystatin C was found to be less erroneous in the estimation of GFR also in elderly [143,144,145,146,147]. Lopes et al. demonstrated that combined creatinine-cystatin C equations had the greatest accuracy, especially in elderly persons, although cystatin C should not replace creatinine in clinical practice: the combination of creatinine and cystatin C appears to provide more accurate GFR estimates in very elderly patients [148]. Moreover, Odden at al. showed that Cystatin C concentration increases significantly with age, even in the absence of risk factors for kidney disease and that particularly in elderly patients, cystatin C is a better marker for estimate GFR, compared with creatinine [149]. Furthermore, among the novel biomarker studied for AKI, NGAL is the most well known. Plasma NGAL starts to elevate within 48-72 h of surgery related AKI, and urinary NGAL elevation significantly predicts adverse outcomes between patients with AKI and sepsis or in critically ill patients seen in ICU [150,151]. In the elderly patients with CKD, serum NGAL has recently emerged to reflect renal impairment and to be closely related to Cystatin C, creatinine, urea, and eGFR. In a cohort of elderly 160 CKD patients (mean age 75.29 ± 12.08 years), Lulu Guo et al. clearly demonstrated that NGAL levels increased progressively with the increase of 2- and 5-year risk of ESRD using the Kidney Failure Risk Equations (KFRE) [152]. Therefore, NGAL can be considered a novel and independent risk marker of kidney function decline in patients aged >50 years and >65 years with advanced CKD. Another previous study from Cullen MR et al. measured NGAL in 174 urine samples from healthy subjects and showed that NGAL concentrations significantly vary with age, in particular the level was higher in 60–88-year-old patients versus 40–59-year-old ones [153]. These studies highlighted the need to normalize the biomarkers cut-off also to age or creatinine, even if this area is still open to debate.

## 7. Prevention and Treatment of AKI in the Elderly

The primary strategy to prevent AKI development in the elderly is to recognize the specific increased vulnerability to renal injury in this cohort of patients [20]. In addition, there are several approaches to reduce the risk of AKI development in this population. The reduction of potentially nephrotoxic drugs intake, such as NSAID, diuretics or aminoglycosides, could help to reduce the AKI development. Furthermore, adequate fluid intake and prevention of hypotensive episode, mainly during invasive procedures, may aid to prevent renal damage [23,38]. Finally, when administration of iodinated contrast media is required for diagnostic or invasive procedures, recent guidelines recommend to reduce as much as possible the volume of iodinated contrast medium and to expand volume with crystalloids and bicarbonate infusion, unless contraindications indicate otherwise [1,154,155,156]. Once AKI is established, there are not specific therapeutic strategies for elderly patients other than those suggested for the general population [1,157]. Mainly, maintenance of renal blood flow and avoidance of further renal injury are the cornerstones of supportive therapies. The decision to start renal replacement therapy (RRT) in the elderly should consider the high risk of dialysis intolerance in these patients and require a coordinated discussion with family members, consulting physicians and other care providers [158]. Several practical guidelines have been designed to support clinical decision-making on the management of older individuals (>65 years of age) with CKD regarding benefits and drawbacks of RRT versus conservative care [159,160]. However, at present, a reduced survival has been shown in the elderly requiring RRT as compared to younger population. As well as in the general population, dialytic therapy in elderly patients with AKI may concur not only to obtain conventional renal replacement but also to treat life-threatening conditions as for sepsis-induced AKI and, in selected cases, to support the residual renal function (Table 2) with optimal nutritional management [161,162].

According to some studies, the age of the patient is not a determining factor for the therapeutic decision-making, which in turn can be influenced by the severity of the lesion, the presence of comorbidities, and the renal functional status [27,163].

Elderly patients could also present increased hemodynamic instability, bleeding [162] and neurological complications which may be due to reduced cardiac function and autonomic dysfunction and to changes in serum osmolality and electrolyte levels. Continuous renal replacement therapy (CRRT) has not been specifically analyzed in the elderly, but it is reasonable that it could grant more stable hemodynamic profile and a lower risk of mild disequilibrium syndrome also in elderly [49,164,165,166].

## 8. Outcome

Patients who develop AKI may recover completely or partially the renal function or may evolve to CKD requiring dialysis or even AKI-related death depending on several factors (comorbidities, sepsis, etc.) [9,167,168].

As compared with the general population, elderly people show limited capacity of recovery and poorer renal condition and survival chances [1]. Although variable among different studies, short-term mortality of elderly patients with AKI is high, ranging between 50% and 75% compared to the younger population [18,20]. These data can be explained by the high degree of severity and complexity of these patients and may vary according to the presence or absence of oliguria, sepsis and multiple organ failure [25,67].

Few studies analyzed the clinical course of elderly patients with AKI in the middle- or long-term. Certainly, elderly patients have higher risk of non-renal recovery after AKI and of progression to CKD [169]. Moreover, AKI development is per se an independent risk factor of long-term mortality in older patients. Bagshaw et al. [166,170] clearly showed that older age was independently associated with increased in-hospital and 1-year mortality in patients with AKI and severe AKI.

However, AKI development seems to impair significantly the renal function recovery in elderly patients. A recent report showed that about 28% of elderly patients aged >65 years did not recover renal function after an episode of AKI, developing CKD afterward because of the lack of compensatory mechanisms and adequate regeneration [25]. Ali T et al. [29] reported that 18.9% of elderly patients with AKI progress to dialysis treatment, and 66.7% of this population die within 1 year.

The preponderance of CKD after AKI in elderly patients should be attributable to the robust fibrotic response that occurs after I/R injury due to AKI episode: this fibrotic response is enhanced by age. The mediators of this relationship are microvascular damage, increased sensitivity to angiotensin II and upregulation of genes associated with inflammation, remodeling and fibrosis [30,103,167,171,172,173].

Finally, it is also important to identify the potential clinical or laboratory predictors of both renal and patient outcome Recently BMI, baseline e GFR, low MAP, low prealbumin level, hypoalbuminemia, oliguria, BUN level and more severe AKI have been shown as independent risk factors associated with poor renal and patient outcomes [169]. The discovery of predictive biomarkers for AKI outcome is crucial in the attempt to early identify and treat this condition and to obtain better clinical outcomes in elderly patients with AKI.

## 9. Conclusions

The mean age of the general population has increased and the healthcare systems are dealing with the increased health demands by the elderly population. AKI is an emerging problem also in the older population, because it often complicates hospitalization, mainly in Intensive Care Unit (ICU) settings, thus significantly worsening morbidity and mortality rates. In the elderly population, the physiological process of “kidney aging”, coupled with the presence of several comorbidities (diabetes, hypertension, heart failure), makes renal tissue more prone to injury and less fit for repair. Considering the emerging relevance of this condition to the internist and emergency setting, a greater attention should be given to the prevention and treatment of AKI in the elderly population in the attempt to improve global outcomes.

## Figures and Tables

**Table 1:** KDIGO classification criteria for acute kidney injury. Abbreviation: KDIGO, Kidney Disease: Improving Global Outcomes.

Stage	Serum Creatinine (SCr)	Urine Output (UO)
1	Baseline increase of 1.5 to 2 times in 7 days	<0.5 mL/kg/h for 6–12 h
2	Baseline increase of 2 to 3 times	<0.5 mL/kg/h for ≥12 h
3	≥4 mg/dL or a baseline increase >3 times or initiation of renal replacement therapy	<0.3 mL/kg/h for ≥24 h or anuria for ≥12 h

**Table 2:** Changes in Aging Kidney. Abbreviations: EGF, epidermal growth factor; GFR glomerular filtration rate; IGF-1, insulin like growth factor 1; VEGF, vascular endothelial growth factor.

Changes in the Aging Kidney
Decrease in total renal mass
Glomerulosclerosis
Thickening of glomerular basement membrane
Thickening of large vessel walls
Decrease in amount and length of tubules
Mesangial expansion
Decrease in renal blood flow (10% for decade from age of 40 years)
Decrease in GFR (1 mL/min/years at age of 45 years)
Blunted nitric oxide production and decreased vasodilatory response
Decreased osmolality
Decreased in renal growth factors production (EGF, IGF-1, VEGF)
Increased susceptibility to cell’s apoptosis
Increased oxidative stress
